# Surgical Treatment of Advanced Thyroid Cancer with Tracheal Invasion

**DOI:** 10.1155/2021/8823405

**Published:** 2021-01-29

**Authors:** Hau Xuan Nguyen, Huy Le Trinh, Hien Xuan Nguyen, Hung Van Nguyen, Quang Van Le

**Affiliations:** ^1^Department of Oncology, Hanoi Medical University, Hanoi, Vietnam; ^2^Department of Oncology and Palliative Care, Hanoi Medical University Hospital, Hanoi, Vietnam; ^3^Department of Head and Neck Surgery, National Cancer Hospital, Hanoi, Vietnam

## Abstract

**Introduction:**

Tracheal invasion in thyroid cancer occurs in one-third of locally advanced cases and is the third most common site of infiltration following strap muscles and recurrent laryngeal nerves. Surgical resection plays an important role in the management strategy followed by either radioactive iodine or external beam radiotherapy. Nonetheless, there has been still controversy about the optimal extension of the surgery. *Case Presentation*. Total thyroidectomy, airway resection and bilateral neck dissection were performed in two cases diagnosed as advanced thyroid cancer with tracheal invasion (stage IV according to McCaffrey). The first case underwent partial tracheal resection and direct anastomosis by the V-shape technique, while the latter one required tracheal resection and permanent tracheotomy. After one-year follow-up, no evidence of tumor recurrence or any postoperative complications were found.

**Conclusion:**

Surgical resection still remains the mainstay of management for advanced thyroid cancer in general and for tracheal invasion cases in particular. The decision of surgical resection and tracheal reconstruction methods mostly depends on the extent of tracheal invasion.

## 1. Introduction

Thyroid cancer is the original from of follicular derived cells or neuroendocrine C-cell-derived types [1]. It is classified into three different histological groups: differentiated thyroid cancer, anaplastic carcinoma, and others (i.e., medullary thyroid cancer). Generally, differentiated thyroid carcinoma (DTC), which accounts for approximately 95% of all thyroid cancers [2], has slow expansion and favorable outcome with 10-year survival over 80% after curative treatment [3]. However, DTC can invade surrounding structures, for instance, strap muscle, recurrent laryngeal nerve (RLN), trachea, and esophagus. Thus, locally advanced DTC is associated with high risks of local recurrence, distal metastasis, and mortality.

The standard treatment of DTC with tracheal invasion is still under controversy. In previous studies, some authors reported that total thyroidectomy and involved structure resection with negative margins could improve survival rates [4–6]. However, others showed that combination therapy including limited operation and radioactive iodine (RAI) therapy or external beam radiotherapy (EBRT) may have similar long-term outcomes and lower risks of surgical complications, compared with aggressive treatment [5, 7]. In addition, some authors have described classification systems for DTC to assess the degree of tracheal invasion [8] and to determine surgical strategy.

In this paper, we reported two cases of DTC invading the trachea with different surgical approaches and favorable outcomes.

## 2. Case Presentation

### 2.1. Case 1

A 54-year-old man with normal previous medical history was admitted to our department because of a mass in his left neck. The patient reported that the tumor had appeared before one year. However, he did not receive any treatment because this tumor did not affect his daily activities. As a result, for the last few months, the tumor had gradually increased in size, and the patient had experienced increasing hoarseness. On examination, there was a 3 × 4 cm, hard and fixed tumor in his left neck without any palpable neck lymph nodes.

**Figure 1:**
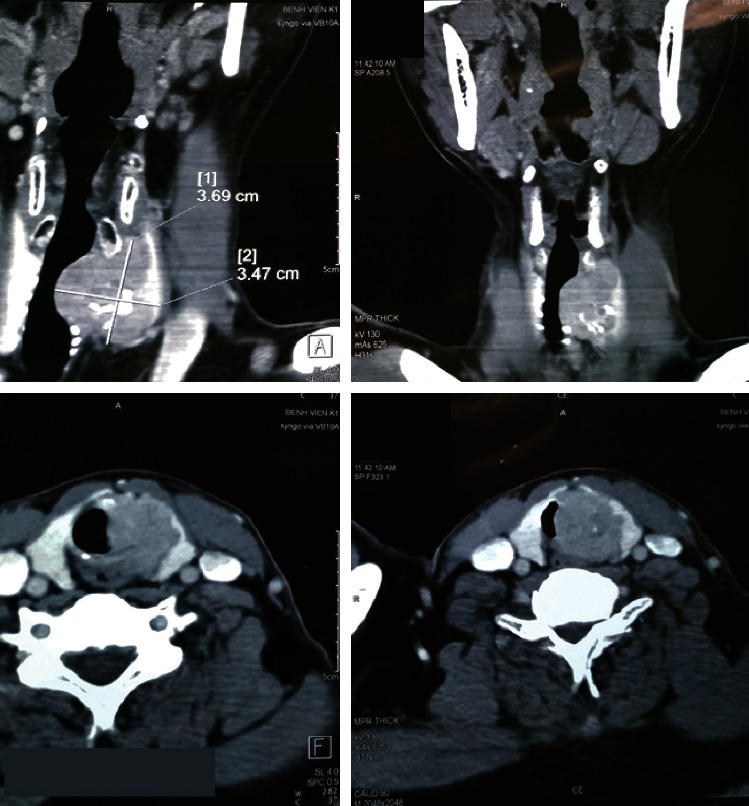
The thyroid tumor compressing the trachea.

Ultrasound suggested a highly suspicious malignant lesion in the left lobe of the thyroid gland, categorized as TI-RADS 5 (according to Thyroid Imaging Reporting and Data System (TI-RADS) 2017). Neck CT scan (as per Figure 1) demonstrated a thyroid tumor with a size of 30 × 37 mm, infiltrating the tracheal wall and compressing the tracheal lumen as well. There were also several small lymph nodes found in the cervical central compartment with the largest diameter below 10 mm. Otorhinolaryngoscopy indicated left vocal cord paralysis, and bronchoscopy showed that tumor is covered by the normal mucosa and narrowing tracheal lumen. Finally, fine needle aspiration concluded papillary thyroid cancer (PTC).

**Figure 2:**
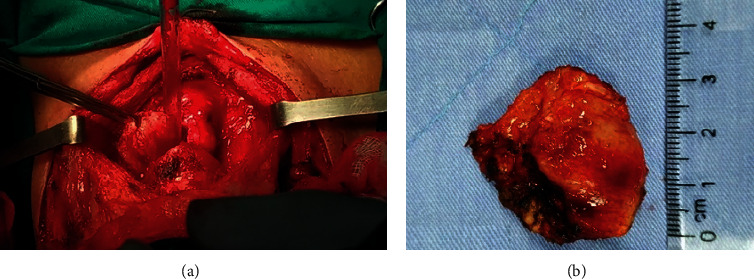
Surgical procedure of partially tracheal resection and end-to-end anastomosis.

Based on the above findings, the patient was diagnosed as left lobe PTC cT4aN0M0, stage I (according to AJCC, 8^th^ edition, 2017) and stage IV (according to McCaffrey). Intraoperative evaluation reported a hard tumor in the size of 30 × 40 mm at the left thyroid lobe, invading the left recurrent laryngeal nerve and the anterior wall of trachea with 30 mm in length. There were multiple lymph nodes in the central compartment and the bilateral neck compartments. The patient underwent total thyroidectomy, central and bilateral cervical lymph nodes dissection, partial tracheal resection, and reconstruction by direct suturing with the V-shape technique (as per Figure 2). The final diagnosis was PTC of the left thyroid lobe staged as pT4aN1bM0. To protect the tracheal anastomosis, the S-head was fixed continuously in the folding position for 6 days. This patient was discharged after 14 days without any complications, and he had his normal neck movement after 1 month. RAI was indicated afterward. During one-year follow-up, there was no evidence of tumor recurrence and postoperative complication.

In addition, the immunohistochemical analysis was performed and revealed that the tumor was positive with CK19, TTF1, CEA (slightly), HBME1, Claudin1, and Ki67 (2%) and negative with CD 56 and p63.

### 2.2. Case 2

A 55-year-old man presented to our hospital with a hard mass in his neck. He noticed that the mass had been gradually increasing in size for six months. In the last month, he reported of having experience of hoarseness and dyspnea. In addition, his previous medical history was unremarkable. Clinical examination showed not only the hard mass with a size of 3 × 4 cm and limited movement but also bilateral cervical lymph nodes.

**Figure 3:**
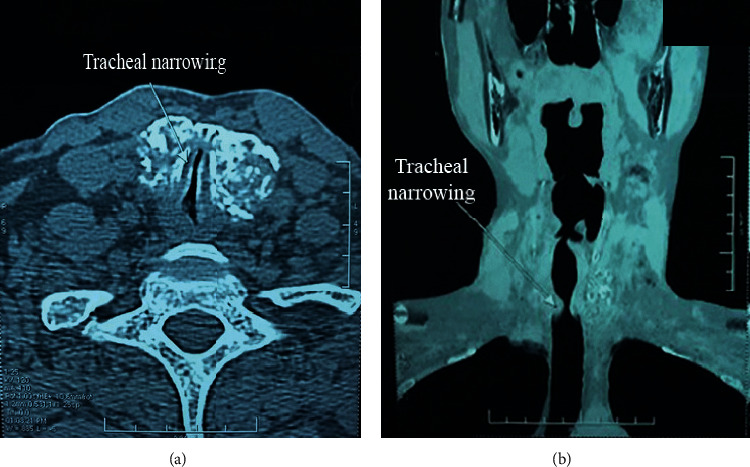
The thyroid tumor compressing the trachea.

Neck ultrasound and 128-slice computed tomography scan (as per Figure 3) revealed a diffusely enlarged thyroid gland, whereas the right lobe size was approximately 16 × 29 mm and the left lobe size was about 28 × 25 mm. The tumor was found to infiltrate the tracheal wall and narrow the tracheal lumen by 90%. Besides, there were suspected metastatic lymph nodes in levels II, III, and IV in both sides with the largest dimension roughly 14 × 10 mm. Flexible fiberoptic laryngoscopy demonstrated limited movement of bilateral vocal cords. Tracheobronchoscopy indicated that the mass invaded the upper part of the trachea but still was covered by normal tracheal mucosa. This mass caused the difficulty in the evaluation of the lower part of the trachea. Other examinations were not particular.

**Figure 4:**
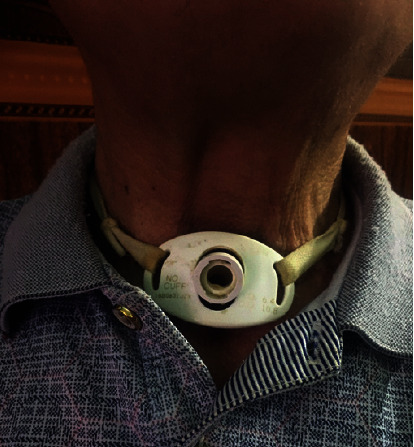
Permanent tracheotomy.

The clinical stage was classified as cT4aN1bM0, stage III (according to AJCC, 8^th^ edition, 2017) and stage IV (according to the classification of McCaffrey et al.). Because endotracheal intubation was not applicable due to constricted trachea, tracheostomy was performed for anesthesia. Intraoperative evaluation has shown that (i) the tumor invaded the trachea in the length of approximately 7 cm and (ii) reconstruction of the remaining parts of the trachea after resection was impractical. Eventually, total thyroidectomy, resection of the involving part of the trachea, and permanent tracheotomy (as per Figure 4) were performed. This patient was discharged after 14 days. Subsequently, RAI ablation was administered. During one-year follow-up, evidence of tumor recurrence and complication was not found.

## 3. Discussion

Generally, diagnosis of thyroid cancer is based on the combination of clinical examination, neck ultrasound, and fine-needle aspiration (FNA). However, in patients with advanced thyroid cancer, it is necessary to perform laryngoscopy, neck CT scan, or neck MRI and sometimes tracheobronchoscopy [9]. Laryngoscopy can detect paralyzed vocal cord cases without voice change. Neck CT scan is used to evaluate the invasion of surrounding structures when tracheobronchoscopy is applied to access the invasive extent of airway lumen. According to Young Lan Seo, CT criteria for trachea invasion include tumor in contact with 180° or more of the tracheal circumferences (grades 3 and 4); deformity of the tracheal lumen at the level of the mass; and focal irregularity, thickening, or bulging in the mucosal portion adjacent to the mass. Based on these criteria, the basis of our criteria, the specificity and accuracy of CT, for tracheal invasion in this study were 91.4% and 83.2%, respectively [10]. However, MRI is more effective than CT in the assessment of invading soft tissue [10]. Most of authors consent to using CT scan for evaluating the tracheal invasion in advanced thyroid cancer [10, 11]. Previous studies showed a significant correlation between superficial lesions in tracheobronchoscopy and the classification of the extent of tracheal invasion [12, 13]. The signs, which suggest the tracheal invasion, include mucosal redness, telangiectasia, mucosal elevation, and mucosal erosion [12]. In our center, three main examinations including laryngoscopy, neck CT scan, and tracheobronchoscopy are regularly applied in case of thyroid carcinoma invading trachea.

The most common staging system applied to evaluate the extent of tracheal invasion in aggressive thyroid cancer is the classification described by Shin and McCaffrey (Table 1). According to McCaffrey, the treatment options of tracheal resection depend on various stages, including total thyroidectomy for stage I, complete gross removal by “shave excision” and total thyroidectomy for stages II and III, and complete tracheal resection for stages III, IV.

Regarding thyroid cancer, tracheal resection could be repaired by the following procedures: (1) shave procedures, (2) segmental/partial resection and direct closure, (3) partial resection together with reconstruction of musculocutaneous flap or cartilage graft or materials, and (4) total laryngectomy and permanent tracheotomy. For stages II and III according to McCaffrey, the resection of all gross tumors can be performed by “shave procedures” meaning the removal of a partial thickness of the airway tract wall. Several retrospective studies comparing radical resection and shave procedures combined with RAI showed no survival benefit in the patients undergoing radical resection [15–18]. Similarly, Segal et al. also reported no difference in 5-year survival between two methods. However, Avenia et al. showed that the overall survival of the completely resected group is better than the positive margins group [6]. For stages IV and V according to McCaffrey, the surgery can be performed by either window resection or circumferential tracheal resection. The first method is appropriate for limited involvement of only anterior or lateral wall of trachea. The defect can be reconstructed by primary closure, with strap muscles, sternocleidomastoid muscle, or latissimus dorsi musculocutaneous flap [19]. Tumor invading extensively the anterolateral tracheal wall can be removed by segmental resection and primary end-to-end anastomosis. The maximum length of segmental tracheal resection is 5-6 cm, which is considered to be adequate for primary anastomosis without tracheal or laryngeal mobilization. In cases that both anastomosis and reconstruction could not be implemented, permanent tracheotomy is inevitable. In our first patient, due to the anterior invasion of the tumor, we decided to remove the anterior wall of 3 tracheal rings and reconstruct the trachea by V-shape. In the second patient, because of the extremely long tracheal segment, we chose the tracheal removal and permanent tracheotomy.

In several previous studies, postoperative complication rate after tracheal resection with direct closure ranges from 15% to 39%, and postoperative mortality rate is roughly 1.2% [20, 21]. The common complications after advanced thyroid carcinoma treatment include anastomotic dehiscence, airway stenosis, infection, and bleeding. The anastomotic dehiscence is one of the most serious complications, which can be life-threatening. This complication is related to the length of the tracheal segment resected over 5-6 cm. To reduce the tension of the anastomotic trachea, the neck of patient is fixed either in a “chin-to-chest” position during the 6^th^ to 7^th^ postoperative day [22–25] or by a C-collar.

## 4. Conclusion

In conclusion, *en bloc* resection of involved trachea is the standard treatment in advanced thyroid carcinoma. The surgical methods are based on the extent of airway wall invasion. The procedure of tracheal resection is safe with low rate of mortality.

## Figures and Tables

**Table 1:** Classification of the extent of tracheal invasion in thyroid cancer.

	Shin et al. [8]	McCaffrey [14]
I	Extension through the capsule of the thyroid gland and abutting the external perichondrium	Tumor locates entirely intrathyroidal gland without airway or surrounding muscle invasion

II	Invasion into the cartilage or the cartilaginous layer or destruction of the cartilage	Tumor invades the perichondrium of the aerodigestive tract or firmly abuts the muscle but does not invade into the cartilage or deeply into muscle

III	Extension into the lamina propria of the tracheal mucosa without epithelial invasion	Tumor invades through the airway perichondrium and into the cartilage or deeply into muscle but not into the submucosa

IV	Invasion into or beyond the trachea	Tumor invades through the perichondrium and cartilage or through the muscle and deforms the submucosa but does not penetrate the mucosa

V		Tumor is gross transmucosal involvement

## Abbreviations

DTC: Differentiated thyroid carcinoma
RLN: Recurrent laryngeal nerve
RAI: Radioactive iodine
EBRT: External beam radiotherapy
TI-RADS: Thyroid imaging reporting and data system
PTC: Papillary thyroid cancer
AJCC: American joint committee on cancer
FNA: Fine-needle aspiration
MRI: Magnetic resonance imaging.
